# Impacts of heavy metal–contaminated feed on growing goats: nutrient digestibility, metal absorption, tissue accumulation, meat quality, and carcass characteristics

**DOI:** 10.3389/fvets.2026.1909059

**Published:** 2026-07-31

**Authors:** Ahmed M. Abd EI Tawab, Shaoxun Tang, Min Gao, Chunyu Jiang, Zhixiong He, Chuanshe Zhou, Zhiliang Tan, Xuefeng Han, Yong-Bin Liu

**Affiliations:** 1 State Key Laboratory of Forage Breeding-by-Design and Utilization, National Engineering Laboratory for Pollution Control and Waste Utilization in Livestock and Poultry Production, and Hunan Provincial Key Laboratory of Animal Nutritional Physiology and Metabolic Process, Institute of Subtropical Agriculture, Chinese Academy of Sciences, Changsha, Hunan, China; 2 Dairy Science Department, National Research Centre, Giza, Egypt; 3 Inner Mongolia Academy of Agricultural & Animal Husbandry Sciences, Hohhot, China; 4 Key Laboratory of Animal Husbandry Science and Technology of Xinjiang Production and Construction Corps, College of Animal Science, Tarim University, Xinjiang, China; 5 College of Advanced Agricultural Sciences, University of Chinese Academy of Sciences, Beijing, China; 6 Department of Animal Genetics, Breeding, and Reproduction, College of Animal Science, Inner Mongolia Agricultural University, Hohhot, China; 7 State Key Laboratory of Reproductive Regulation & Breeding of Grassland Livestock, Inner Mongolia University, Hohhot, China

**Keywords:** carcass characteristics, digestibility, goats, heavy metals, meat quality

## Abstract

**Introduction:**

In areas affected by heavy metal contamination, the potential transfer of these metals from feed into animal products during digestion is a major concern.

**Methods:**

Twenty-four Xiangdong black male goats were equally assigned to three experimental groups (8 goats per group) using a completely randomized design for 60 days. The experimental groups were formulated by incorporating Paddy A, B, and C (containing low [LHM], medium [MHM], and high [HHM] levels of heavy metals, respectively) at 33% of the total diet.

**Results:**

The results revealed a nonsignificant decrease (*p* > 0.05) in the digestibility and absorption of Mn, Ca, Fe, Zn and Cr through the gastrointestinal tract, whereas the absorption of Cd and Pb significantly increased (*p <* 0.05), and that of Ni decreased in the HHM and MHH groups compared with those in the control group. The contents of heavy metals and essential metals in different tissues changed according to the type and function of the organ, and the contents of heavy metals in various tissues (spleen, lung, liver, kidney, rumen, ileum, cecum, colon, rectum, fat, muscles and hair) remained within internationally recognized safety limits for human consumption, as established by Codex and/or the European Commission Regulation. Moreover, meat quality and carcass characteristics were not affected (*p* > 0.05) by increasing heavy metal contents in the diets of goats.

**Conclusion:**

This study demonstrates that, under the current experimental conditions, incorporating paddy cultivated in soils contaminated with heavy metals into Xiangdong black male goat diets for short periods (60 days), without adverse effects on productive performance or health status, and the resulting animal products remained safe for human consumption. However, long-term studies involving different animal species under diverse climatic conditions are required to validate these findings before considering any practical application in livestock production systems.

## 1 Introduction

Livestock production is a cornerstone of the global economy, yet disease-associated losses account for over 20% of productivity declines worldwide (1). Beyond infectious agents, heavy metal toxicity particularly from cadmium (Cd), lead (Pb), and nickel (Ni) presents a critical non-infectious threat to ruminant health (2–4). Industrial and commercial activities have led to widespread contamination of soil, water, plants, and grains (5, 6). When ruminants consume contaminated feed, heavy metals accumulate in tissues, disrupt gut microbiota, impair nutrient absorption, and cause multi-organ damage, while also promoting long-term antibiotic resistance (7–9).

Although the gastrointestinal tract absorbs heavy metals, a substantial proportion remains in the intestinal tract, where it may affect the gut microbiota and functionality (10). In ruminants, exposure to Pb and Cd disrupts gut microbiota specifically increasing *Lactobacillaceae* and *Erysipelotrichaceae* while suppressing *Lachnospiraceae* which impairs short-chain fatty acid production and triggers intestinal inflammation, enteritis, and colitis (11, 12). Moreover, heavy metals, such as Cd, Pb, Cr, Cu, Hg, Ni and As, have a negative effect on the rumen microbiome, such as *Ruminococcus albus, Bacteroides succinogenes, Eubacterium ruminantium,* and *Bacteroides amylophilus,* which are strongly related to the feed digestibility (7, 10).

Several studies have highlighted the impact of heavy metal contamination of feed on animal productivity and health (8, 10, 13–15). Ruminants such as cattle, goats, and sheep are exposed to heavy metal via contaminated feed, which can impair nutrient absorption, compromise gut health, and accumulate in tissues, potentially leading to toxicity and posing risks to human health through the food chain (16). Compared with cattle, goats show greater tolerance to toxic metals and excrete larger amounts of them in their feces (17). Systemically, chronic exposure to heavy metals disrupts core metabolic processes, leading to reduced feed intake, poor body condition, and diminished growth (18). Cd exposure, for instance, has been shown to reduce the digestibility of dry matter and crude protein in ruminants (19). Heavy metal contamination in animal feeds can interfere with nutrient absorption and adversely affect meat quality (13). Milk can, also be contaminated with toxic substances such as Pb, Hg, and Cd (20). Furthermore, these toxic contaminants can bioaccumulate in animal tissues and may subsequently enter the human food chain, posing a significant threat to public health (7, 14, 15).

Heavy metals can damage multiple organs and systems in animals, including kidneys, liver, and nervous, cardiovascular, respiratory, reproductive, digestive, and endocrine systems, leading to organ damage, dysfunction, and disease (21, 22). Common heavy metals associated with kidney injury at high exposure levels include Pb, As, and Cd (23, 24). Additionally, these metals are carcinogenic, mutagenic, and teratogenic, and can induce immunosuppression, poor body conditions, and decreased reproduction performance in domestic animals (25).

Due to widespread heavy metal contamination of agricultural land in regions like China, evaluating the viability and safety of using crops grown in these soils as livestock feed is critical. Therefore, this study aimed to assess the impact of incorporating rice paddies cultivated in heavy metal-contaminated soils (Cd, Pb, and Ni) into the diet of Xiangdong black male goats for short periods, and to evaluate the consequent impact on nutrient digestibility, essential metals absorption, heavy metals accumulation in meat and edible offal, meat quality, and carcass characteristics.

## 2 Materials and methods

### 2.1 Study site

The experiment was carried out at the Animal Research Facility of the Institute of Subtropical Agriculture, Changsha, China. The area has an annual average rainfall of approximately 1,795 mm and a mean annual temperature of about 18.3 °C, with seasonal variations ranging from cold winters (around 6 °C in January) to hot summers (up to 33 °C in July).

### 2.2 Goats and feeding management

Twenty-four healthy Xiangdong black male goats with a similar body weight of 14.1 ± 1.39 kg, an average age of 9 months, and the same genetic background were randomly selected into three groups (*n* = 8 goats/group) in a complete randomized design. Goats were individually kept in single cages (0.80 × 1.20 m/goat) with free access to drinking water, fed according to the National Research Council (NRC) (26) recommendations. The goats were fed a basal diet; the total mixed ration (TMR) consists of 33% paddy A, 9.6% soy bean meal, 6% wheat bran, 3.2% fat powder, 0.5% calcium carbonate, 1.1% calcium bicarbonate, 0.6% sodium chloride, 1% premix^1^, and rice 45% straw as a control group (LHM). In other treatments, goats were fed a control diet with paddy A replaced by paddy B (MHM) or paddy C (HHM). The rice paddy was cultivated in soils contaminated with varying levels of heavy metals, and was used to prepare three diets with different heavy metal levels. The heavy metals contaminated rice paddy was provided by the Beishan test station (Changsha, China), which is located in a contaminated zone. The quantity of heavy metals in the rice paddy was determined before its use in this study (Table 1). The ingredients and nutritional composition of experimental diets are shown in Table 1. The experiment lasted for 60 days for the real trial and 14 days for adaptation. Goats were fed twice daily at 07:00 and 19:00 h. The behavioral and health status of the goats was monitored during feeding. The daily temperature and humidity inside the house were recorded, and the goat house was cleaned daily.

**Table 1:** Ingredients, nutrient compositions and metal contents of experimental diets.

Item	LHM	MHM	HHM	SEM	*p* value
Ingredient % of DM					
Paddy A	33	–	–	–	–
Paddy B	–	33	–	–	–
Paddy C	–	–	33	–	–
Soy bean meal	9.6	9.6	9.6	–	–
Wheat bran	6	6	6	–	–
Fat powder	3.2	3.2	3.2	–	–
Calcium carbonate	0.5	0.5	0.5	–	–
Calcium bicarbonate	1.1	1.1	1.1	–	–
Sodium chloride	0.6	0.6	0.6	–	–
Premix^1^	1	1	1	–	–
Rice straw	45	45	45	–	–
Nutrient composition g/kg					
DM	880.5	885.4	882.1	5.27	0.945
OM	881.3	886.2	883.2	17.51	0.995
CP	96.4	95.3	94.8	4.73	0.993
EE	46.7	45.2	44.7	3.15	0.973
NDF	480	478	476	7.48	0.982
ADF	338	334	333	7.86	0.972
Ash	118.7	113.8	116.8	4.87	0.937
Metals content mg/kg					
Mn	46.35	46.11	46.79	0.440	0.853
Ca	7,549.1	7,287.1	7,291.2	77.89	0.329
Fe	473.7^a^	388.8^c^	414.3^b^	12.81	<0.0001
Zn	15.13^a^	11.32^b^	11.51^b^	0.654	0.001
Cr	1.30	1.01	1.09	0.057	0.077
Cd	0.23^c^	0.63^b^	1.07^a^	0.121	<0.0001
Pb	0.23^c^	0.28^b^	0.35^a^	0.017	<0.0001
Ni	0.50^b^	0.78^ab^	1.09^a^	0.098	0.017

Premix per kg; Mn: 5.1 g; Mg: 8.5 g; Fe: 510 mg; Se: 5 mg; Zn: 4 g; Cu: 750 mg; I: 30 mg; Co: 11 mg; vitamin A: 104,400 IU; vitamin E: 2,200 IU and vitamin D: 17,500 IU. LHM, low heavy metals; MHM, medium heavy metals; HHM, high heavy metals. Paddy A, low heavy metals content; paddy B, medium heavy metals content; paddy C, high heavy metals content. DM, dry matter; OM, organic matter; CP, crude protein; EE, ether extract; NDF, neutral detergent fiber; ADF, acid detergent fiber. Mn, Manganese; Ca, calcium; Fe, iron, Zn, zinc; Ni, nickel; Cr, chromium; Cd, cadmium, and Pb, lead.

### 2.3 Feed intake and sampling of fecal and urine

Feed intake was determined by subtracting the amount of feed and orts provided from the previous day’s feeding. During the last 7 days of the experiment, the digestibility trial was conducted using the total collection technique; the total feces and urine produced each day were weighed and recorded for each animal. 10% of the total feces was taken and dried at 55 °C for 72 h, crushed by a grinder, and stored at room temperature. Also, 10% of the urine sample was stored at −20 °C. TMR was collected and dried as described by (27).

### 2.4 Sampling of blood plasma

Blood samples (5 mL) were collected on the 60th day of each experimental period for all goats in the morning at 4 h post-feeding from the jugular vein of goats into vacuum-sealed blood collection tubes with sodium heparin as an anticoagulant (Aosaite Medical Devices Co., LTD, Heze, China). Each blood sample was centrifuged at 3,000 g at 4 °C for 10 min to separate the plasma into 2 mL Eppendorf tubes and then stored at −20 °C for metals detection.

### 2.5 Tissue samples, carcass and meat quality

At the end of the experiment period, all goats were slaughtered by a licensed veterinarian after 16 h of fasting with free access to water, and body weight was measured before the animals were slaughtered. After slaughtering, the internal viscera such as liver, spleen, lung, kidney, digestive tract (rumen, ileum, caecum, colon and rectum), and fat, and the external offal such as four feet, head, hair and skin were separated for each animal. The empty bodies were cut in half across the spinal column. Carcasses and viscera were weighed. The samples of spleen, lung, kidney, liver, rumen, ileum, cecum, colon, rectum, fat, and muscles were freeze-dried and then ground into a powder to determine the metal concentrations.

### 2.6 Samples analysis

#### 2.6.1 Chemical composition analysis of feed, feces, and tissue samples

The collected samples were subjected to analytical measurements of dry matter (DM) by method no. 934.01, ether extract (EE) by method no. 920.39, crude protein (CP) by method no. 954.01, and ash by method no. 942.05 (28). Fiber fraction (acid detergent fiber (NDF) and neutral detergent fiber (ADF)) contents were determined in a Fibretherm FT 12 Fiber Analyzer (C. Gerhardt GmbH & Co. KG) using the methods described by Van Soest et al. (29). Prior to NDF analysis, samples were treated with sodium sulfite and alpha-amylase. There is no ash residue when NDF and ADF are expressed. Organic matter (OM) was calculated as OM g/kg = 1,000 − ash content.

Perinephric fat, Gluteus Medius, Vastus lateralis muscle, longissimus dorsi muscle, and the loin eye area were determined as described by Lima et al. (30). Three more pieces of 2 × 2 cm longissimus dorsi muscle were taken for pH and meat color measurements at 45 min, the sample was stored at 4 °C for 24 h, and tested again by pH meter. The meat colors (L, a, and b) were measured as described by Majdoub-Mathlouthi et al. (31) and Santos et al. (32).

#### 2.6.2 Determination of metals

Feed, blood plasma, fat, muscles, and offal samples were digested with concentrated nitric acid (HNO3) and diluted with double-distilled water, as described by Yao et al. (33), with minor modifications. Briefly, the weighed samples were transferred to triangular flasks containing 15 mL of acid mixture (HNO3: HClO4 = 5:1(v/v)). The flasks were covered with funnels and left at room temperature for cold digestion overnight. Then, the digestion flasks were placed on the electric heating panel. Digestions were initially performed at 120 °C until the solutions turned yellow transparent, and then, the temperature was increased to 180\u00B0C for 30 min. The temperature was further increased to 260 °C until the solutions turned colorless. Then, the funnels were removed from the flasks, and the solutions were almost completely evaporated. After cooling to room temperature, the residues were dissolved in 1% (v/v) HNO3 and transferred into a 10 mL colorimetric tube, and the final volume was made up to 10 mL. After filtration, the concentrations of metals in the digested samples were determined by inductively coupled plasma optical emission spectroscopy (ICP-OES 700 Series, Agilent Technologies, USA). The blank digests without samples and the reference material were also treated using the same protocol to confirm the accuracy of the analytical results. The concentrations of metals were calculated on a dry weight basis, and the results are expressed as milligrams per kilogram.

## 3 Statistical analysis

The data were analyzed by analysis of variance using the GLM procedure of the SAS software package (SAS OnDemand for Academics, SAS Inst., Inc., Cary, NC). Unless stated otherwise, all the data are presented as the means ± standard deviations (±SDs) or standard error means ±SEM. Comparisons among groups were analyzed via a complete randomized design followed by Duncan’s test. The significance threshold was set at *p <* 0.05.


Yijk=μ+Mi+Lj+(ML)ij+eijk

Where: Yijk: observed response (e.g., meat quality, heavy metals concentration, digestibility rate), *μ* is the overall mean, Mi: effect of heavy metal type (Pb, Cd, Ni), Lj: effect of heavy metal level (low, medium, high), (ML)ij: interaction between type and level, eijk: experimental error.

## 4 Results

### 4.1 Feed intake, nutrient digestibility, and metal absorption in goats

The effect of dietary heavy metals on nutrient digestibility and metal absorption in goats is presented in Table 2. No significant differences were observed in feed intake or the digestibility of DM, OM, CP, EE, NDF, and ADF (*p* = 0.130, *p* = 0.296, *p* = 0.661, *p* = 0.948, *p* = 0.449, and *p* = 0.893, respectively) among groups. Similarly, the absorption of essential metals (Mn, Ca, Fe, Zn, and Cr) did not differ significantly (*p* = 0.596, *p* = 0.303, *p* = 0.259, *p* = 0.801, and *p* = 0.173, respectively) between goats fed HHM diets and the control group. In contrast, the absorption of Ni in the gut gradually decreased (*p* < 0.0001) with increasing levels of heavy metal contamination in the diet. While, the absorption of Cd and Pb increased sharply under medium and high contamination (*p <* 0.0001, and *p* = 0.005, respectively).

**Table 2:** Effect of feeding heavy metal-contaminated feed on feed intake, nutrient digestibility and metal absorption in goats.

Item	LHM	MHM	HHM	SEM	*p* value
Feed intake	561.0	543.2	514.2	13.12	0.367
Nutrient digestibility (g/kg)					
DM	605.3	603.8	588.1	3.85	0.130
OM	659.6	657.3	646.5	3.63	0.296
CP	541.9	537.6	525.7	7.34	0.661
EE	687.6	685.9	678.2	12.13	0.948
NDF	608.6	583.0	581.7	9.61	0.449
ADF	511.7	506.0	500.5	9.32	0.893
Metals absorption (%)					
Mn	24.10	24.45	22.27	0.913	0.596
Ca	54.48	51.65	50.43	1.083	0.303
Fe	31.05	26.16	26.33	1.362	0.259
Zn	36.13	36.73	35.65	0.637	0.801
Cr	6.99	6.02	6.06	0.245	0.173
Cd	5.85^b^	7.09^a^	7.26^a^	0.126	<0.0001
Pb	6.65^b^	7.11^a^	6.97^a^	0.062	0.005
Ni	9.38^a^	9.30^b^	8.96^c^	0.036	<0.0001

Means in the same row with different letters (a, b, and c) differ (*p* < 0.05). SEM: standard error means.

LHM, low heavy metals; MHM, medium heavy metals; HHM, high heavy metals. DMD, dry matter digestibility; OMD, organic matter digestibility; CPD, crude protein digestibility; EED, ether extract digestibility; NDFD, neutral detergent fiber digestibility; ADFD, acid detergent fiber digestibility. Mn, Manganese; Ca, calcium; Fe, iron, Zn, zinc; Ni, nickel; Cr, chromium; Cd, cadmium, and Pb, lead.

### 4.2 Blood plasma chemistry

The concentrations of metals in blood plasma were affected by the level of heavy metal contamination in the feed (Table 3). Plasma concentrations of Mn, Ca, Fe, and Zn were significantly reduced (*p* < 0.0001, *p* = 0.015, *p* = 0.004, and *p* = 0.008, respectively) in goats fed highly contaminated feed compared with those fed low and medium contaminated diets. No significant differences were observed for Cr (*p* = 0.544) and Ni (*p* = 0.108). In contrast, Cd and Pb concentrations were significantly higher in the MHM and HMH groups compared with the LHM group (*p* = 0.003, and *p <* 0.0001, respectively).

**Table 3:** Effect of feeding heavy metal-contaminated feed on plasma metal concentration.

Item	LHM	MHM	HHM	SEM	*p* value
Metals concentration μg/ml					
Mn	0.448^a^	0.415^a^	0.218^b^	0.018	<0.0001
Ca	156.67^a^	156.86^a^	146.95^b^	1.64	0.015
Fe	10.23^a^	9.62^ab^	8.59^b^	0.192	0.004
Zn	3.56^a^	3.54^a^	3.42^b^	0.017	0.008
Cr	0.444	0.438	0.416	0.010	0.544
Cd	0.008^b^	0.010^a^	0.011^a^	0.0004	0.003
Pb	0.163^b^	0.253^a^	0. 268^a^	0.011	<0.0001
Ni	0.176	0.169	0.147	0.005	0.108

Means in the same row with different letters (a, b, and c) differ (*p* < 0.05). SEM, standard error means.

LHM, low heavy metals; MHM, medium heavy metals; HHM, high heavy metals.

Mn, Manganese; Ca, calcium; Fe, iron, Zn, zinc; Ni, nickel; Cr, chromium; Cd, cadmium, and Pb, lead.

### 4.3 Metal contents in internal organ tissues

**Figure 1:**
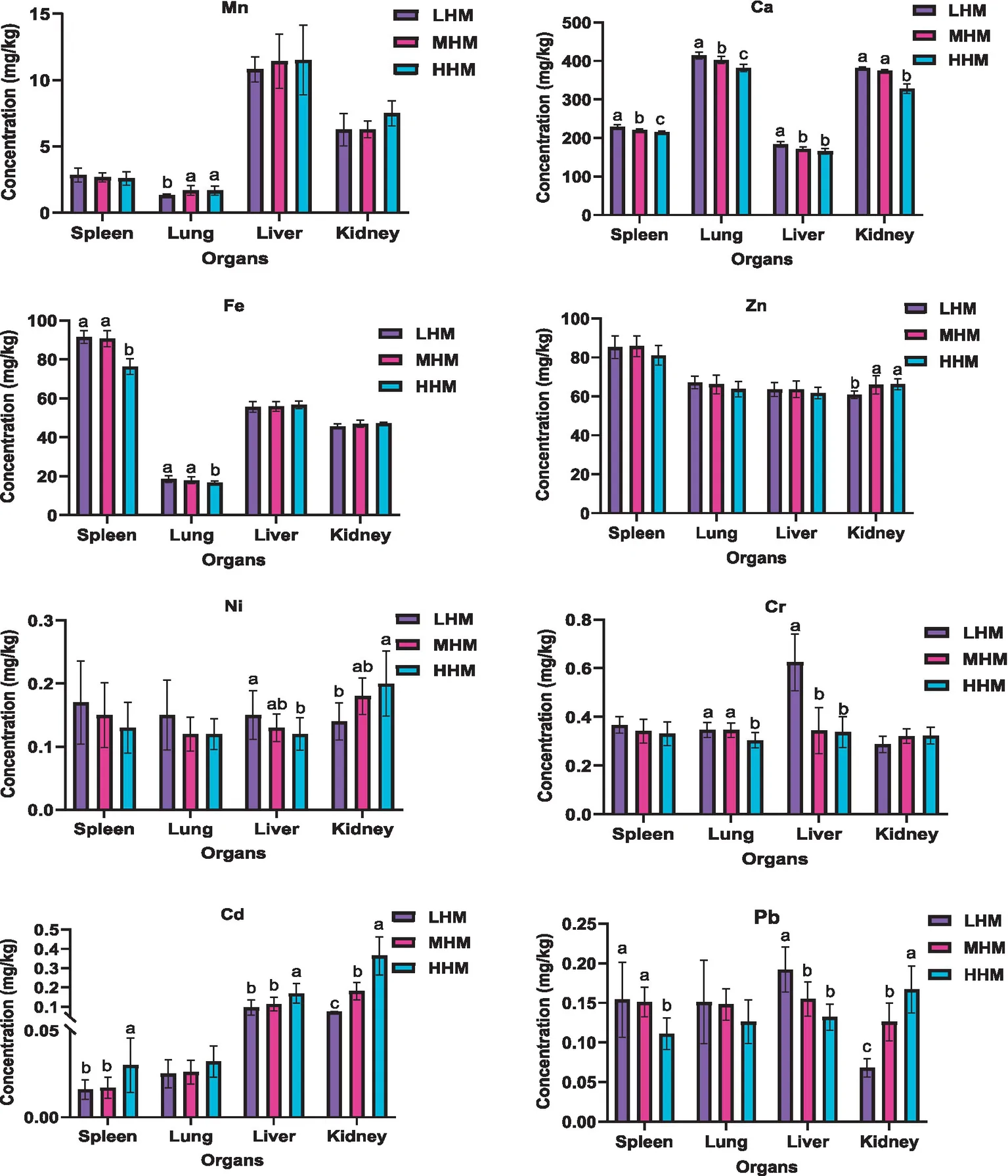
Effect of feeding heavy metal contaminated feed on the metals content of internal organs tissues in goats. LHM, low heavy metals; MHM, medium heavy metals; HHM, high heavy metals. Mn, manganese; Ca, calcium; Fe, iron; Zn, zinc; Ni, nickel; Cr, chromium; Cd, cadmium; and Pb, lead.

The effect of dietary heavy metals on metal contents in internal organs (spleen, lung, liver, and kidney) is shown in Figure 1. In spleen tissues, Ca, Fe, and Pb concentrations decreased significantly (*p* > 0.05) as the level of heavy metals in goat diets increased, while Cd increased significantly (*p* > 0.05). No significant differences were observed for Mn, Zn, Ni, and Cr. In lung tissues, Mn increased significantly (*p* > 0.05) with increasing dietary heavy metals, while Ca, Fe and Cr decreased significantly (*p* > 0.05). Zn, Ni, Cd, and Pb showed no significant changes. In liver tissues, Cd significantly increased (*p* > 0.05) with increasing levels of heavy metals in the diets, while Ca, Cr, Ni and Pb reduced (*p* > 0.05). No significant differences were detected in Mn, Fe, and Zn among groups. In kidney tissues, Zn, Ni, Cd and Pb increased significantly (*p* > 0.05) in goats fed MHM and HHM diets, while Ca decreased significantly (*p* > 0.05) compared with the control. Fe, Cr, and Mn showed no significant changes.

### 4.4 Metal contents in digestive tract tissues

**Figure 2:**
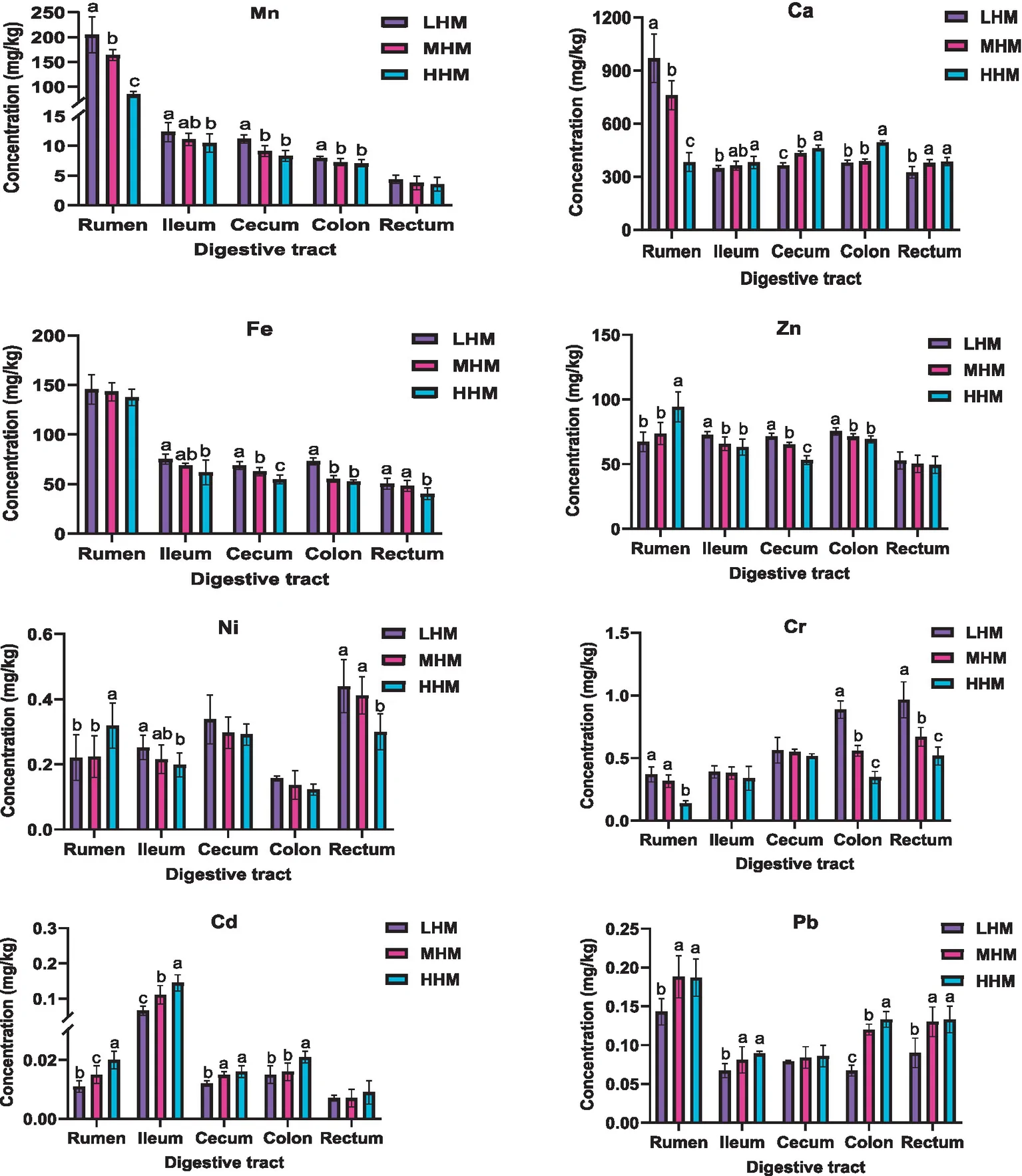
Effect of feeding heavy metal contaminated feed on the metals content of digestive tract tissues in goats. LHM, Low heavy metals; MHM, Medium heavy metals; HHM, High heavy metals. Mn, Manganese; Ca, Calcium; Fe, Iron, Zn, Zinc; Ni, Nickel; Cr, Chromium; Cd, Cadmium, and Pb, Lead.

In general, Figure 2 illustrates the concentrations of metals (Mn, Ca, Fe, Zn, Ni, Cr, Cd, and Pb) in different sections of the digestive tract (rumen, ileum, cecum, colon, and rectum) of goats under varying levels of dietary heavy metals contamination. In rumen tissues, Mn, Ca, and Cr concentrations were highest in goats fed low heavy metals and decreased significantly in the HHM group (*p* < 0.05),while Fe showed no significant differences. In contrast, Cd and Pb concentrations were significantly higher (*p* < 0.05) in the MHM and HHM groups compared with the control. Interestingly, Zn and Ni exhibited the opposite trend, with their concentrations increasing in rumen tissues under heavy metal exposure (*p* < 0.05). In ileum and cecum tissues, Mn, Fe, Zn, Ni, and Cr concentrations decreased significantly with increasing dietary heavy metals (*p* < 0.05), whereas Ca, Cd, and Pb concentrations increased significantly in the MHM and HHM groups (*p <* 0.05). In colon tissues, Mn, Fe, Zn, and Cr concentrations decreased significantly (*p <* 0.05) with increasing dietary heavy metal levels, while Ca, Cd and Pb concentrations increased (*p <* 0.05). Ni showed no significant differences. In rectum tissues, Fe, Cr, and Ni concentrations were lowest (*p* < 0.05) in the HHM group compared to the LHM group, confirming the overall trend of decreasing concentrations of essential metals. In contrast, Ca and Pb concentrations increased significantly (*p* < 0.05), while Mn, Zn, and Cd showed no significant changes.

### 4.5 Metal contents in fat, muscles and hair tissues

**Figure 3:**
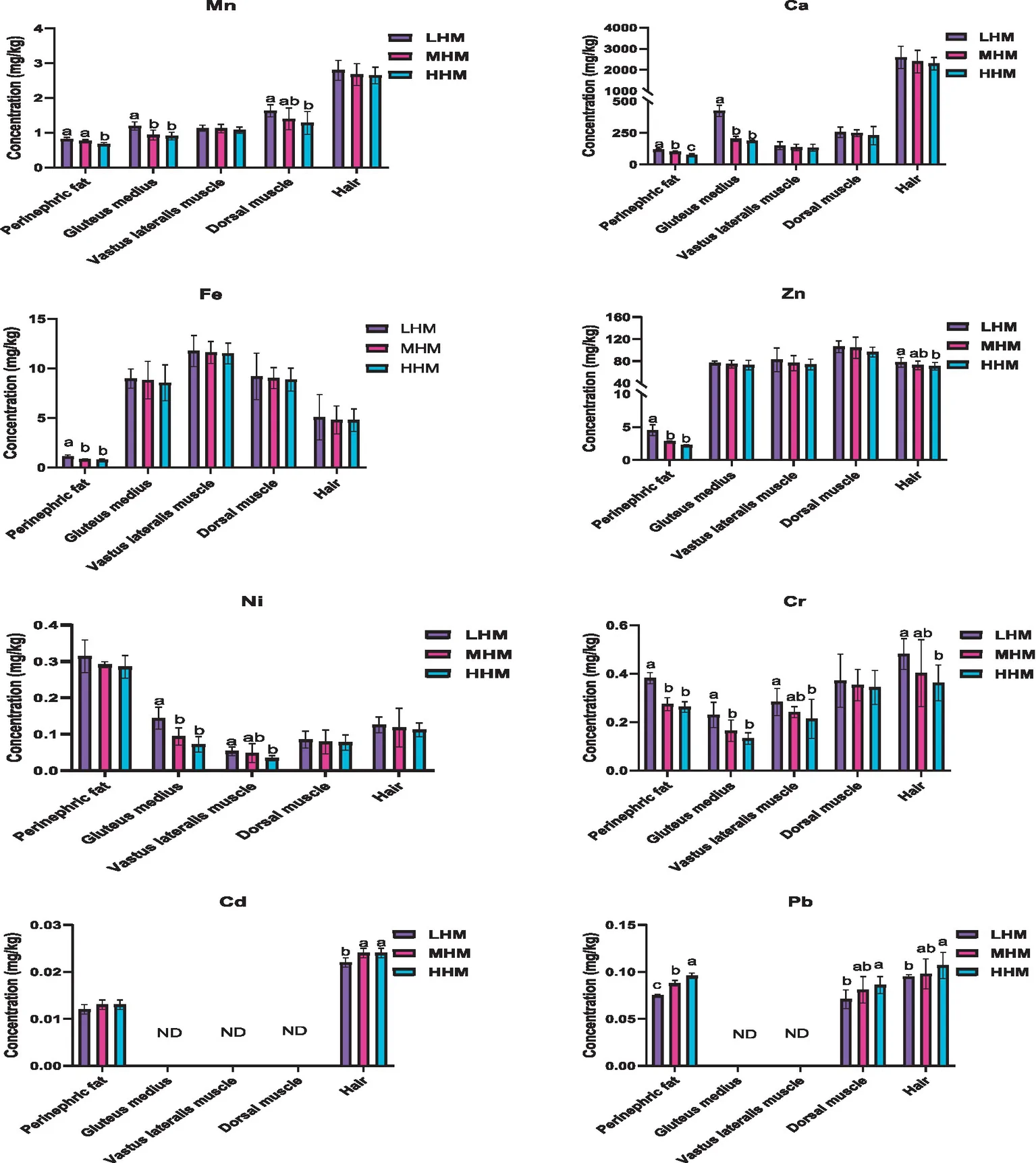
Effect of feeding heavy metal contaminated feed on the metals content of fat, muscles, and hair tissues in goats. LHM, low heavy metals; MHM, medium heavy metals; HHM, high heavy metals. Mn, Manganese; Ca, Calcium; Fe, Iron; Zn, Zinc; Ni, Nickel; Cr, Chromium; Cd, Cadmium, and Pb, Lead.

The concentrations of metals (Mn, Ca, Fe, Zn, Ni, Cr, Cd, and Pb) in perinephric fat, *gluteus medius, vastus lateralis muscle, dorsal muscle*, and hair tissues of goats are presented in Figure 3. In perinephric fat, Mn, Ca, Fe, Zn, and Cr concentrations were highest (*p* < 0.05) in the LHM group and decreased significantly in the MHM and HHM groups. Ni and Cd showed no significant changes (*p* > 0.05) among groups, while Pb concentrations exhibited marked increases (*p* < 0.05) in the MHM and HHM groups compared with the LHM group. In muscle tissues, Mn, Ca, Ni, and Cr concentrations decreased in the MHM and HHM groups. Pb concentrations increased in the *dorsal muscle* (*p <* 0.05), while no Pb was detected in the *gluteus medius* or *vastus lateralis muscles.* No significant changes were observed in Fe and Zn levels (*p* > 0.05). Moreover, cadmium (Cd) was not detected in muscle tissues. In hair tissues, Cd and Pb concentrations were significantly higher in the MHM and HHM groups compared with the LHM (*p* < 0.05). Cr and Zn concentrations decreased significantly in the MHM and HHM groups (*p* < 0.05), while Mn, Ca, Fe, and Ni showed no significant differences.

### 4.6 Carcass characteristics and meat quality

**Figure 4:**
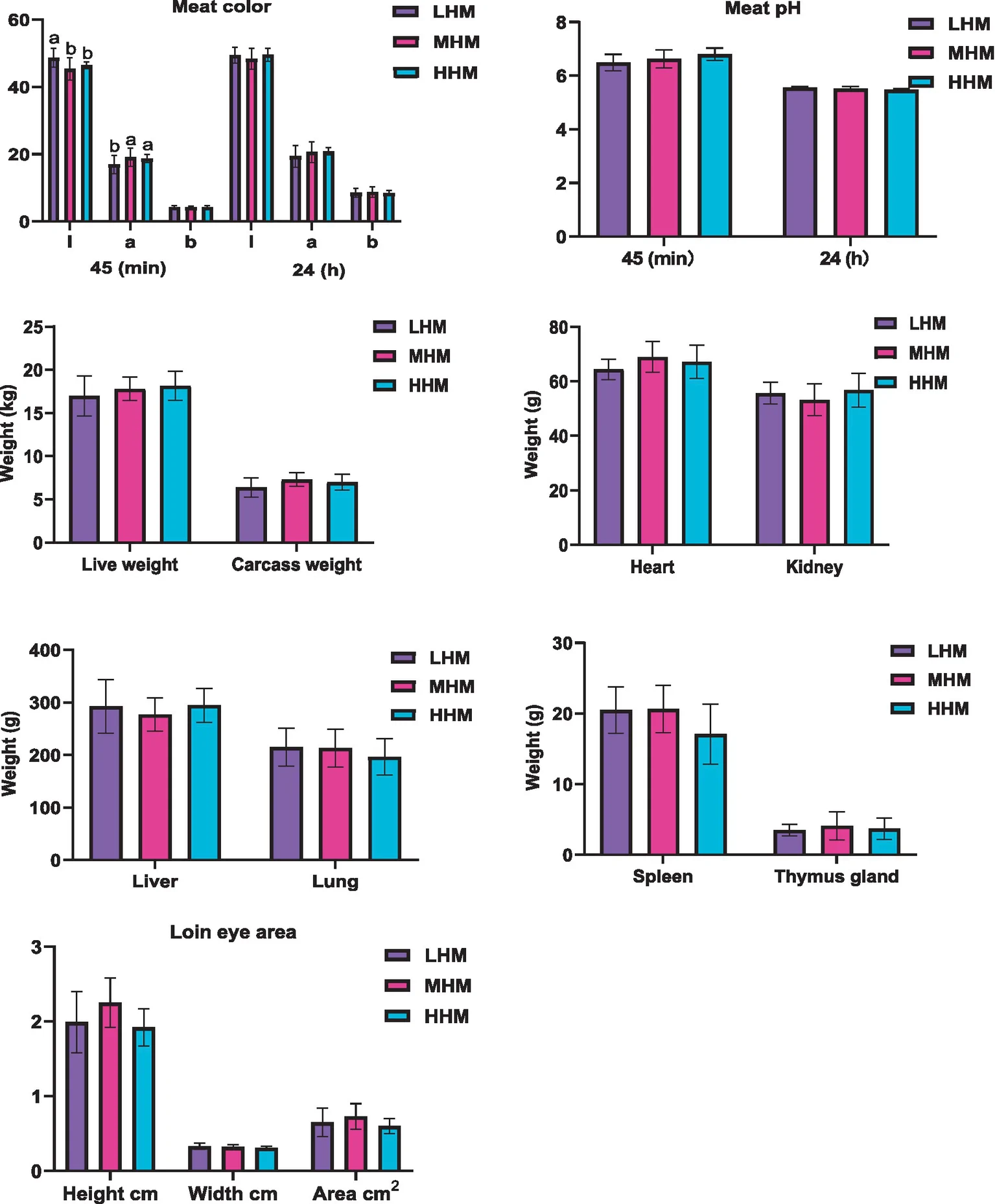
Effect of feeding heavy metal contaminated feed on meat quality and carcass characteristics in goats. LHM, low heavy metals; MHM, medium heavy metals; HHM, high heavy metals.

Figure 4 shows the effect of dietary heavy metals on carcass characteristics and meat quality. No significant differences (*p* > 0.05) were observed in live body weight, carcass weight, or the weights of liver, spleen, lung, kidney, and thymus among groups. Similarly, loin eye area and meat pH at 45 min and 24 h did not differ significantly (*p* > 0.05). At 45 min post-slaughter, muscle brightness (L*) decreased significantly (*p <* 0.05), carcass redness (a*) increased significantly (*p <* 0.05), while yellowness (b*) showed no significant differences among groups. No significant differences were observed among groups for *L, a,* or b* values at 24 h.

## 5 Discussion

Dietary contamination with heavy metals is the main source of exposure for livestock in agricultural areas (18). These metals can accumulate in animal feed and manure, presenting risks to both animal health and food safety (14, 34). In our study, feed intake and nutrient digestibility were not significantly affected at moderate or high levels of heavy metals. Similar outcomes were reported by Salem (35), who observed that feeding sheep cadmium-contaminated feed at a level of 5 mmol/L in drinking water for 3 months resulted in a slight reduction in feed intake and impaired nutrient digestibility. Also, Xu et al. (16) found that there was no significant decrease in DMI in beef cattle fed Cd contaminated diets (6.74 mg/kg) for 107 days. During a 60-day trial with Egyptian Nubian goats, exposure to drinking water containing 0.91 mg/L Pb and 0.304 mg/L Cd disrupted rumen fermentation and reduced VFA production, lowering DMD (36). In goats, low nickel levels (1–10 ppm) for several months show little direct effect on feed intake or digestibility (37). Goats and sheep are able to detoxify and eliminate heavy metals efficiently, which explains the absence of significant changes in feed intake (4). The absorption of Mn, Ca, Fe, Zn and Cr was reduced at medium and high levels of heavy metals in the diet, this reflects the ability of goats to regulate essential metal absorption even under contamination (4). Moreover, some metals can bind to heavy metals, preventing their absorption and reducing their impact on the goat’s system (38, 39). Ni absorption decreased compared with other metals, consistent with reports that goats excrete more toxic metals in feces than cattle (17).

Decreased plasma essential metal concentrations at high levels of dietary heavy metals were associated with decreased absorption into the gastrointestinal tract (Table 2). In addition, heavy metals can disrupt the function of transport proteins responsible for absorption, leading to decreased plasma metal levels (40). Antunović et al. (41) reported decreases in the concentrations of Ni, Cr, and Cd in the blood of goats fed contaminated diets (0.29, 0.57, and 0.04 mg/kg, respectively), after 90th day compared with 30th day of sampling. Also, Ogabiela et al. (42) observed increased in Cd and Pb concentrations in the blood of cows fed contaminated diets (0.17 ppm Cd and 0.79 ppm Pb) over a long-term period.

Dietary exposure to heavy metals can affect the distribution of metals in various tissues of goats (43, 44). In our study, calcium concentrations decreased significantly in spleen, lung, liver, and kidney with increasing dietary heavy metals, the decrease attributed to heavy metals can compete with calcium for binding sites (45). Muktar et al. (44), similary reported reductions in Ca internal organs under heavy metal exposure (Pb and Cd). Mn content increased in lung tissues, which may reflect changes in binding proteins and transport competition (46, 47). Fe concentrations decreased in spleen and lung tissues with high heavy metals in the goat diet likely due to disruption of transporters such as transferrin and ferroportin and displacement from binding sites, leading to decreased levels of free Fe in blood plasma (Table 2) and tissues (Figure 1) (44, 48). The research findings are consistent with those of Muktar et al. (44), who found that heavy metals such as Pb and Cd can cause Fe deficiency in the internal organs. Increased concentration of Zn, Ni, Cr and Pb in kidney tissues at the high levels of heavy metals in goat diets, reflecting the kidney’s role in filtering and accumulating metals (49). In contrast, the concentration of these metals decreased in the spleen, liver and lung tissues, as Cd content increased with increased heavy metals in goat diets. This pattern is consistent with competition between Cd and essential metals for intestinal transporters and interference with protein-mediated delivery (50). In addition, Cd can interfere with the functions of transport proteins to carry metals in the bloodstream, and this effect can hinder the delivery of Zn, Ni, Cr and Pb to various organs, including the spleen, liver, and lungs. Gebeyew et al. (51) reported similar increases in Cd in liver and kidney tissues of goats fed contaminated diets (1.07 mg/kg DM for 60 days). Furthermore, Tsuneishi et al. (52) noted that there was an increase in Cd content observed in the liver and kidneys, and to a lesser extent in the spleen and lungs, with rising Cd intake as CdCl₂·2.5H₂O in beef cattle.

Since the functions of each part of the digestive system differ, metal concentrations vary across tissues depending on the element and contamination level. In our study, Mn, Fe and Cr contents decreased significantly in gastrointestinal tissues with increased levels of heavy metals in goat diets, through various mechanisms, including alteration of gut microbiota, competition for absorption, formation of insoluble complexes, and interference with transport (53, 54). Pb can affect the activity of enzymes involved in heme synthesis and can interfere with Fe absorption, while Cd competes for the same transporter, divalent metal transporter 1 (9, 53). Zn and Ni concentrations lowered in the ileum, caecum, colon, and rectum, while they increased in the rumen tissues with increased heavy metals in the goat diet. This reflects direct interactions with heavy metals forming insoluble compounds (Table 2), reduced intestinal absorption, and differences in pH, microbial activity, and retention time between rumen and intestine (55, 56). Ca content increased in intestinal tissues but decreased in rumen tissue under high contamination, likely due to insoluble Ca complexes formed with Pb and Cd, which reduce soluble calcium available for absorption (57). Cd and Pb concentrations increased in digestive tract tissues in parallel with dietary levels (58).

Concentrations of Mn, Ca, Zn, Ni and Cr in fat, muscles and hair tissues decreased with increased heavy metals in goat diets, consistent with reduced plasma concentrations of these elements (Table 3). Competition between heavy metals and essential metals for absorption in the gastrointestinal tract reduces the absorption of Mn, Ca, Zn, Ni and Cr (Table 2), resulting in lower tissue concentrations (59). Cd content was not detected in muscle tissue, while fat and hair content increased, and Pb content in fat, *dorsal muscle* muscle and hair tissue increased with increasing heavy metals in goat diets. These results are consistent with those of Gebeyew et al. (51), who reported that Cd content was not detected in muscle tissue at high levels of Cd in goat diets (1.07 mg/kg) for 60 days. Also, Tsuneishi et al. (52) observed that Cd content was not detected in muscle or hair tissues with rising cadmium intake as CdCl₂·2.5H₂O in beef cattle. At the same time, Salgado-Souto et al. (60) found increased Cd and Pb in goat hair fed a contaminated diet (153 and 118 μg/kg DM of Pb and Cd, respectively) for 7 months. Moreover, Patra et al. (61) confermed a relationship between dietary heavy metals and their content in the muscles or hair tissues. Su et al. (62) noted increased Cd content in the hair of cows fed a contaminated diet containing cadmium chloride at a level of 0.182 mg/kg body weight per day for 21 days. Lee and Lee (63) observed that the concentrations of metals such as Cd, Zn and Fe in the blood and their contents were reflected in the hair of rats.

The results in this study showed no significant effect on live body weight, carcass, liver, spleen, lung, kidney and thymus, and no change in eye area and meat pH at 45 min and 24 h between groups exposed to different levels of heavy metals. The absence of significant changes may be related to the relatively short duration of the experiment, which limited heavy metal accumulation, and to the ability of goats to adapt to contamination (19). Xu et al., (16) also reported no changes in weight gain in beef cattle fed cadmium-contaminated diets (6.74 mg/kg) for 107 days. In contrast, Spears and Hatfield, (64) reported an increase in live body weight gain in lambs fed on Ni-contaminated diets at a level of 5 ppm for 28 days.

Color is believed to be the primary visual cue that influences customers’ choices and evaluations of meat quality (65). Heavy metals decreased brightness (L*) by 6.8 and 4.4 for MHM and HHM, respectively. This means the meat tends to be darker than the LHM. However, the L* values recorded for these treatments exceeded the minimum (≥ 34) for consumer acceptance (66). Heavy metals increased redness (a*) of meat from 10.6–12.6%, which suggests that the antioxidant components may be linked to increased red pigment and stability. According to Simitzis et al. (67) and Luciano et al. (68), consumers will accept meat with an a* value of 9.5 or higher.

The concentrations of Cd, Pb, and Ni in goat meat and edible offal tissues were evaluated against internationally recognized food safety standards. The average maximum values obtained (Cd: 0.00 and 0.364 mg/kg; Pb: 0.00 and 0.192 mg/kg; Ni: 0.144 and 0.440 mg/kg, on a dry weight basis for meat and edible offal tissues, respectively) were lower than the maximum levels established by COMMISSION REGULATION (EC) No 1881/2006, (69) for Cd (0.05 mg/kg for meat and 0.5 mg/kg for offal) and for Pb (0.10 mg/kg for meat and 0.5 mg/kg for offal) fresh weight basis. In contrast, no maximum levels have been established for Ni in meat or edible offal under Codex Alimentarius Commission (the issuing body) (70), given its comparatively lower toxicological concern relative to Cd and Pb. Based on these reference criteria, the detected concentrations fall within acceptable safety margins and do not pose an immediate risk to human consumption. Nevertheless, from a public health perspective, continuous monitoring remains essential, as chronic exposure to Cd and Pb may pose long-term risks, and Ni, though unregulated, warrants attention for its potential health effects.

## 6 Conclusion

In the present study, heavy metal contamination in goat diets had no significant effect on feed intake and nutrient digestibility. Furthermore, the accumulation of heavy metals in internal organs (spleen, liver, lung, kidney), gastrointestinal tissues (rumen, ileum, cecum, colon, rectum), and fat, muscles and hair, but concentration in meat and edible offal remained within the internationally recognized safety limits for human consumption according to Codex Alimentarius, 1995 and/or European Commission Regulation (EC) No 1881/2006, 2006. Moreover Meat quality and carcass characteristics were not affected by dietary contamination. Therefore, the findings indicate that, under the specific contamination levels tested and within the 60-day feeding period, goat diets containing heavy metals did not adversely affect productive performance or health. However, the conclusion is limited to the short period and the contamination levels applied, and caution is warranted before generalizing the safe use of contaminated paddy as feed under different conditions or longer exposure periods.

## Data Availability

The raw data supporting the conclusions of this article will be made available by the authors, without undue reservation.
